# Study on the clinical outcomes of COVID-19 infection and immune responses following the third booster dose of inactivated COVID-19 vaccine in hematologic disease patients

**DOI:** 10.3389/fpubh.2026.1917493

**Published:** 2026-10-27

**Authors:** Haiying Zhang, Xin Wang, Runling Zhang, Feng Liu, Hongsong Chen, Yuyuan Jia, Huiying Rao

**Affiliations:** 1 Infectious Disease and Hepatology Center of Peking University People’s Hospital, Peking University People’s Hospital, Peking University Hepatology Institute, Beijing, China; 2 State Key Laboratory of Natural and Biomimetic Drugs, Peking University, Beijing, China; 3 Peking University Third Hospital, Beijing, China

**Keywords:** allogeneic hematopoietic stem cell transplantation, COVID-19, hematologic diseases, inactivated COVID-19 vaccine, neutralizing antibodies, SARS-CoV-2 RT-PCR

## Abstract

**Background:**

Patients with hematologic diseases may have impaired immunity owing to their underlying diseases and immunosuppressive treatments. Evidence on documented SARS-CoV-2 RT-PCR outcomes and responses to inactivated COVID-19 vaccines remains limited.

**Methods:**

This retrospective study analyzed three independently identified populations: 20,813 individuals tested by SARS-CoV-2 RT-PCR, 2,438 tested for anti-SARS-CoV-2 IgG, and 1,946 adults tested after two or three vaccine doses. Intervals to documented RT-PCR negativity were analyzed only among positive individuals with subsequent negative results. Vaccination analyses were adjusted for age, sex, testing interval, and disease group. Monthly comparisons were cross-sectional. Serial antibody measurements were analyzed separately.

**Results:**

Recorded RT-PCR positivity was lower among tested individuals with hematologic diseases (4.77% vs. 10.74%; *p* < 0.001). Among 64/64 positive individuals with and 381/2,091 without hematologic diseases who had documented subsequent negative results, median intervals to negativity were 5.05 versus 3.99 days (difference, 1.06 days; 95% CI, 0.73–2.21; *p* < 0.001). Within the hematologic disease group, longer intervals were observed after prior immunosuppressive treatment (5.99 vs. 3.97 days); haploidentical allogeneic hematopoietic stem cell transplantation (allo-HSCT) recipients had the longest descriptive median interval (6.97 days). In the separate IgG-testing population, positivity did not differ significantly (86.00% vs. 90.16%; *p* = 0.174). Three versus two doses were associated with higher neutralizing antibody (NAb) positivity (94.1% vs. 77.3%; adjusted prevalence ratio [aPR], 1.281; 95% CI, 1.232–1.332) and IgG positivity (97.2% vs. 83.4%; aPR, 1.229; 95% CI, 1.189–1.271). Among 20 paired participants, 19 had higher NAb levels after the third dose. Among three-dose recipients, NAb positivity was 25/32 (78.13%) with hematologic diseases, 36/42 (85.71%) with solid tumors, and 743/780 (95.26%) without documented hematologic disease or solid tumors (overall *p* < 0.001).

**Conclusion:**

Among RT-PCR-positive individuals with documented subsequent negativity, intervals were modestly longer with hematologic diseases. Within this group, median intervals were longer after immunosuppressive treatment, and longest among haploidentical allo-HSCT recipients. Differential availability of negative results warrants caution. Three inactivated vaccine doses were associated with higher NAb and IgG positivity overall. Among three-dose recipients, NAb positivity was lower with hematologic diseases than without documented hematologic disease or solid tumors.

## 1 Introduction

SARS-CoV-2, the virus responsible for the COVID-19 pandemic, has infected over 770 million people (1), and can lead to severe pneumonia, acute respiratory distress syndrome (ARDS), multiple organ dysfunction syndrome, and even death (2–5), with approximately 7 million fatalities (1). Although the peak of the pandemic has passed, SARS-CoV-2 is highly likely to persist long-term as an endemic virus due to the cumulative mutations under selective pressure and its enhanced ability to evade immunity (6–8). Therefore, identifying and protecting high-risk susceptible populations remains a central task in current public health and clinical interventions.

Patients with hematologic disorders are affected by their underlying conditions, such as disruption of the bone marrow hematopoietic microenvironment, and previous exposure to complex treatments including chemotherapy, targeted therapies, immunosuppressants, or allogeneic hematopoietic stem cell transplantation (allo-HSCT), resulting in profound or persistent immunodeficiency states (9). Available evidence indicates that this population not only faces an approximately 19% higher risk of SARS-CoV-2 infection compared to the general population but also exhibits significantly greater morbidity and mortality burdens post-infection (10, 11). Real-world data demonstrate a 28-day mortality rate of up to 50.7% among hematologic disease patients hospitalized with COVID-19 (12). A large retrospective study in Spain revealed that patients with hematologic diseases have twice the risk of developing severe COVID-19 compared to the general population (13). Among vaccinated patients with hematologic diseases, the risk of poor prognosis nearly doubles, even without active chemotherapy (14). Additional studies show that 23.5% of hematologic disease patients co-infected with COVID-19 developed severe/critical illness despite a vaccination rate of 97.6%, with a total COVID-19 mortality rate of 22.4% and a 30-day mortality rate of 11.8% (15). These findings suggest that conventional prevention strategies and routine vaccination may fail to provide adequate protection for immunocompromised hematologic disease patients, who represent a high-risk group requiring prioritized attention in ongoing COVID-19 containment efforts.

Vaccination against COVID-19 is a key approach to inducing specific immunity and reducing severe disease and mortality rates (16, 17). However, current studies on neutralizing antibody (NAb) responses to the third dose of inactivated COVID-19 vaccines in real-world settings, as well as on vaccine responses and clinical outcomes in immunocompromised populations, remain significantly limited. The study by Parai et al. only analyzed antibody responses following vaccination with BBV-152 and AZD1222 in the Indian population, lacking targeted data from China (18). The study by Zeng et al. relied solely on clinical trial data and failed to reflect real-world vaccination efficacy (19). Although Liang et al. focused on the Chinese population, their sample size was small, limiting the generalizability of their findings (20). More critically, existing studies have demonstrated weakened immune responses and lower seroconversion rates in patients with hematologic diseases after vaccination (21). Additionally, most available studies lack detailed data covering diverse populations, including those with hematologic diseases or solid tumors, and the patterns of vaccine responses in immunocompromised individuals remain poorly understood.

To address these gaps, we conducted a single-center retrospective study in three independently identified populations. Among individuals who underwent SARS-CoV-2 RT-PCR testing, we compared recorded positivity by hematologic disease status. Among those with a positive result and a documented subsequent negative result, we compared the interval to documented RT-PCR negativity. In a separate IgG-testing population, we compared antibody positivity by hematologic disease status. In vaccinated adults, we compared NAb and IgG responses after two or three doses of an inactivated vaccine and examined responses across disease groups. Serial antibody measurements were analyzed separately where available. Together, these analyses aim to provide real-world evidence on documented RT-PCR outcomes and vaccine-associated humoral responses. They may help frame monitoring priorities and inform prospective studies of booster responses in patients with hematologic diseases.

## 2 Methods

### 2.1 Study design and data source

This single-center retrospective observational study was conducted at Peking University People’s Hospital using routinely collected laboratory data, electronic medical records, and official vaccination records. Three analytic populations were independently identified: (1) individuals who underwent SARS-CoV-2 reverse-transcription polymerase chain reaction (RT-PCR) testing; (2) individuals who underwent serum anti-SARS-CoV-2 IgG testing; and (3) adults who underwent serological testing after receiving two or three doses of an inactivated SARS-CoV-2 vaccine. The three populations were analyzed separately and did not represent sequential stages of a single cohort. The vaccination and antibody-testing population constituted the primary population for evaluating vaccine-associated antibody responses. The selection of participants for each analysis is shown in Figures 1A,B.

**Figure 1:**
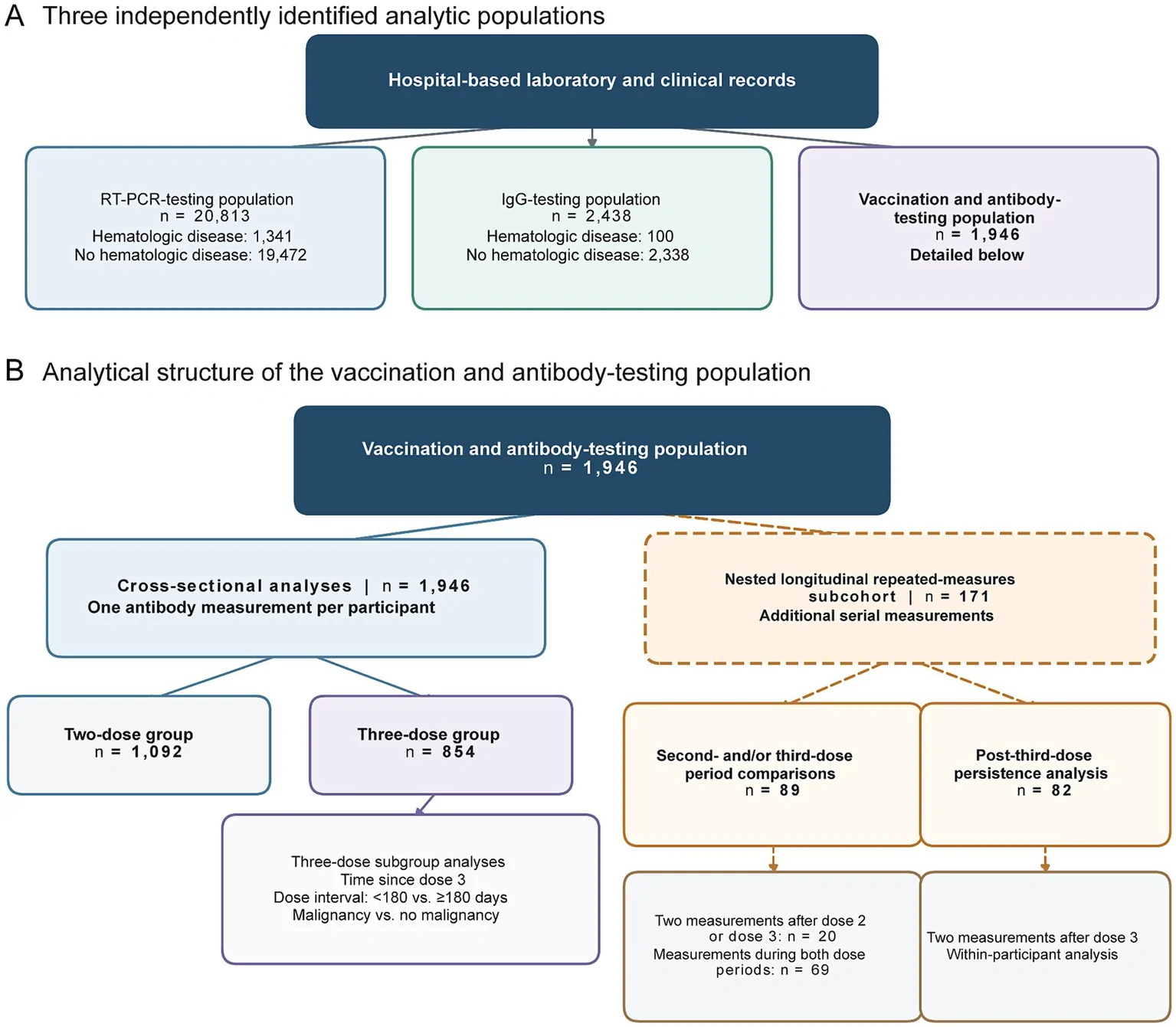
Flow diagram of participant selection and analytic populations. **(A,B)** The diagram shows the selection of the SARS-CoV-2 RT-PCR-testing population, anti-SARS-CoV-2 IgG-testing population, vaccination and antibody-testing population, and repeated-measures subcohort. RT-PCR, reverse-transcription polymerase chain reaction; IgG, immunoglobulin G.

### 2.2 Study populations

#### 2.2.1 SARS-CoV-2 RT-PCR-testing population

The RT-PCR-testing population included 20,813 individuals who underwent SARS-CoV-2 RT-PCR testing between October 2023 and September 2025. Based on diagnoses recorded in the electronic medical records, participants were classified as having (*n* = 1,341) or not having (*n* = 19,472) a hematologic disease. SARS-CoV-2 RT-PCR test positivity was compared between the two groups (Table 1).

**Table 1:** SARS-CoV-2 RT-PCR results according to hematologic disease status.

Variable	Hematologic disease (*n* = 1,341)	No hematologic disease (*n* = 19,472)	*χ^2^*	Effect estimate (95% CI)	*p*-value
Positive RT-PCR result, *n* (%)	64 (4.77)	2,091 (10.74)	48.11	RR, 0.44 (0.35–0.57)	<0.001
Negative RT-PCR result, *n* (%)	1,277 (95.23)	17,381 (89.26)			
Interval to documented RT-PCR negativity, days (*n* = 64 and *n* = 381)	5.05 (3.26–10.66)	3.99 (2.95–5.03)		Difference in medians, 1.06 days (95% CI, 0.73–2.21)	<0.001

RR, risk ratio. Denominators represent individuals who underwent RT-PCR testing; the RR refers to recorded RT-PCR positivity in this tested population.

The analysis of the interval to documented RT-PCR negativity included only individuals with a subsequent negative result (64 with and 381 without hematologic diseases). Individuals without a documented subsequent negative result were excluded, and no observations were treated as censored.

Among individuals with a positive RT-PCR result, the index date was the date of the first positive result during the study period. The interval to documented RT-PCR negativity was calculated from the index date to the first subsequent negative result. If two consecutive negative results at least 24 h apart were available, the date of the first negative result was used; if only one subsequent negative result was available, its date was used. This analysis included only individuals with a documented subsequent negative result: 64 of 64 RT-PCR-positive individuals with hematologic diseases and 381 of 2,091 without hematologic diseases. Individuals without a documented subsequent negative result were excluded from this analysis.

Because the RT-PCR-testing population was identified from routine laboratory records, detailed demographic and clinical information was not uniformly available for all tested individuals. More detailed clinical data were therefore collected only for RT-PCR-positive patients with hematologic diseases, including age, sex, hematologic diagnosis, immunosuppressive treatment before infection, allo-HSCT status at the index date, and clinical outcomes. The distribution of hematologic diagnoses is presented in Tables 2 and 3.

**Table 2:** Clinical characteristics and outcomes of RT-PCR-positive patients with hematologic diseases according to allo-HSCT status.

Characteristic	HLA-matched allo-HSCT (*n* = 15)	Haploidentical allo-HSCT (*n* = 30)	No prior allo-HSCT (*n* = 19)	Overall (*n* = 64)
Demographic characteristics				
Age (years)	40 (31.0–55.0)	36 (32.5–58.5)	33 (28.0–53.0)	37 (30.3–55.8)
Male sex, *n* (%)	7 (46.67)	18 (60.00)	14 (73.68)	39 (60.94)
Han ethnicity, *n* (%)	15 (100.00)	30 (100.00)	17 (89.47)	62 (96.88)
Hematologic diagnosis, *n* (%)				
Acute myeloid leukemia (AML)	7 (46.67)	12 (40.00)	10 (52.63)	29 (45.31)
Acute lymphoblastic leukemia (ALL)	3 (20.00)	7 (23.33)	6 (31.58)	16 (25.00)
Myelodysplastic syndrome (MDS)	2 (13.33)	6 (20.00)	1 (5.26)	9 (14.06)
Chronic myelomonocytic leukemia (CMML)	2 (13.33)	0	0	2 (3.13)
Chronic myeloid leukemia (CML)	0	2 (6.67)	0	2 (3.13)
T-lymphoblastic lymphoma (T-LBL)	0	2 (6.67)	0	2 (3.13)
Plasma cell leukemia (PCL)	0	0	2 (10.53)	2 (3.13)
Aplastic anemia (AA)	1 (6.67)	0	0	1 (1.56)
Myeloid sarcoma (MS)	0	1 (3.33)	0	1 (1.56)
Treatment characteristics				
Immunosuppressive treatment before SARS-CoV-2 infection, *n* (%)	15 (100.00)	29 (96.67)	2 (10.53)	46 (71.88)
Immunosuppressive treatment initiated after infection, *n* (%)	NR	1 (3.33)	13 (68.42)	NR
Clinical outcomes				
Interval to documented RT-PCR negativity, days	4.77 (3.11–11.15)	6.97 (4.87–14.30)	4.00 (2.90–6.19)	5.05 (3.26–10.66)
Death during follow-up, *n* (%)	0	2 (6.67)	1 (5.26)	3 (4.69)

Data are presented as *n* (%) unless otherwise indicated. Allo-HSCT, allogeneic hematopoietic stem cell transplantation; NR, not reported; RT-PCR, reverse transcription polymerase chain reaction. Transplantation status was determined at the time of the index positive RT-PCR result.

The AML group included one patient who initially presented with hyperleukocytosis.

**Table 3:** Interval to documented RT-PCR negativity according to prior immunosuppressive treatment.

Prior immunosuppressive treatment	Patients, (*n*)	Patients included in time-to-negativity analysis, *n*	Interval to documented RT-PCR negativity, Median (IQR), days	*p*-value
Yes	46	46	5.99 (4.03–11.35)	0.010
No	18	18	3.97 (2.93–5.32)	

Prior immunosuppressive treatment refers to documented systemic immunosuppressive use within 12 months before the first positive RT-PCR result.

Prior immunosuppressive treatment was defined as documented use of systemic immunosuppressive agents within the 12 months preceding the first positive SARS-CoV-2 RT-PCR result. Included agents were systemic corticosteroids, cyclosporine, mycophenolate mofetil, and methotrexate or antithymocyte globulin used in immunosuppressive regimens.

#### 2.2.2 SARS-CoV-2 IgG-testing population

The SARS-CoV-2 IgG-testing population included 2,438 individuals who underwent serum anti-SARS-CoV-2 IgG testing at Peking University People’s Hospital between October 2022 and September 2025. Based on diagnoses recorded in the electronic medical records, participants were classified as having a hematologic disease (*n* = 100) or not having a hematologic disease (*n* = 2,338). The proportion of participants with a positive anti-SARS-CoV-2 IgG result was compared between the two groups.

#### 2.2.3 Vaccination and antibody-testing population

The primary vaccination and antibody-testing population included 1,946 adults aged ≥18 years who underwent SARS-CoV-2 antibody testing at Peking University People’s Hospital between January 19, 2021, and March 3, 2022, and had received two or three doses of an inactivated SARS-CoV-2 vaccine. Participants with a documented history of SARS-CoV-2 infection or a positive RT-PCR result before antibody testing were excluded. Vaccination status and vaccination dates were obtained from official vaccination records. According to the number of vaccine doses completed at the time of blood sampling, 1,092 participants were classified into the two-dose group and 854 into the three-dose group. The vaccines included in the study were manufactured by the Beijing Institute of Biological Products Co., Ltd. and the Wuhan Institute of Biological Products Co., Ltd.

Age, sex, underlying diseases, vaccine manufacturer, vaccination dates, intervals between vaccine doses, and the interval between the most recent vaccine dose and antibody testing were recorded. The interval between the two most recent vaccine doses reported in refers to the first-to-second dose interval in two-dose recipients and the second-to-third dose interval in three-dose recipients. For the cross-sectional analyses, each participant contributed one antibody measurement. NAb positivity and levels were compared between the two-dose and three-dose groups. Anti-SARS-CoV-2 IgG positivity and positivity for either anti-SARS-CoV-2 IgM or IgG were also compared between the groups.

Participants were also categorized according to the number of months between the most recent vaccine dose and blood sampling. These time categories comprised participants tested at different post-vaccination intervals, and the number of participants varied across months. Among three-dose recipients, antibody responses were additionally compared across three groups: hematologic diseases, solid tumors, and no documented hematologic disease or solid tumor. Analyses were also stratified according to the interval between the second and third vaccine doses, categorized as <180 days or ≥180 days.

A longitudinal repeated-measures subcohort comprised 171 participants with serial antibody measurements. Follow-up schedules were not uniform, and participants did not necessarily contribute measurements at all assessment points. Of these, 89 participants contributed measurements during the second- and/or third-dose vaccination periods. Only 20 had measurements available after both the second and third doses and were included in the direct paired comparison; the remaining 69 did not contribute a complete second- versus third-dose pair. The remaining 82 participants underwent antibody testing at two different time points after the third dose and were included in the within-participant analysis of post-booster antibody responses. Only participants with measurements available at both time points relevant to a given comparison were included in the corresponding paired analysis. Serial measurements were analyzed separately and were not treated as independent observations in the cross-sectional analyses.

### 2.3 Laboratory examination

#### 2.3.1 SARS-CoV-2 RT-PCR testing

Pharyngeal swab specimens were collected for SARS-CoV-2 nucleic acid testing. Nucleic acid extraction was performed using nucleic acid extraction reagents (Changzhou Jinmaige Biotechnology Co., Ltd.). SARS-CoV-2 RNA was detected by real-time reverse-transcription PCR using the SARS-CoV-2 (2019-nCoV) nucleic acid detection kit manufactured by Daan Gene Co., Ltd. of Sun Yat-sen University, in accordance with the manufacturer’s instructions.

### 2.4 Measurement of SARS-CoV-2 antibodies

Serological tests were performed using the Autolumo A2000 Plus fully automated chemiluminescent immunoassay analyzer (Antu Bioengineering Co., Ltd., Zhengzhou, China). All assays were performed according to the manufacturers’ instructions using reagent kits approved by the China National Medical Products Administration (NMPA).

Anti-SARS-CoV-2 IgM/IgG antibodies were measured using a SARS-CoV-2 IgG antibody detection kit (Wantai Kairui Biotechnology Co., Ltd., Xiamen, China) based on the capture method, along with corresponding reagents. The particle surface was coated with anti-human IgM/IgG antibodies, which then bind to horseradish peroxidase (HRP)-labeled SARS-CoV-2 antigens for detection.

NAb levels were measured using a competitive chemiluminescence immunoassay based on the inhibition of binding between the SARS-CoV-2 receptor-binding domain (RBD) and angiotensin-converting enzyme 2 (ACE2) (22). Results were expressed in AU/mL.

### 2.5 Statistical analysis

Statistical analysis was performed using SPSS 21.0 (IBM Corp., Armonk, NY, United States) and GraphPad Prism 9.0 software (GraphPad Software, San Diego, CA, United States). Bootstrap confidence intervals were calculated using Python version 3.12 with SciPy version 1.17.0. Antibody levels were log10-transformed before parametric testing and linear regression analysis, as appropriate.

Continuous variables were assessed for normality using the Shapiro–Wilk test. Normally distributed variables were presented as mean ± standard deviation (SD) and were compared using the independent-samples *t*-test; Welch’s correction was applied when the homogeneity-of-variance assumption was not met. Non-normally distributed variables are presented as median and interquartile range (IQR) and were compared using the Mann–Whitney *U* test. Categorical variables were presented as numbers and percentages and were compared using the Pearson χ^2^ test or Fisher’s exact test, as appropriate.

For the comparison of RT-PCR positivity, an unadjusted risk ratio with a 95% confidence interval was calculated, with individuals without hematologic diseases serving as the reference group. The interval to documented RT-PCR negativity was summarized as the median and interquartile range among individuals with a subsequent negative result. The two groups were compared using the Mann–Whitney *U* test. The between-group effect was expressed as the difference in medians, calculated as the median interval in individuals with hematologic diseases minus that in individuals without hematologic diseases. Its 95% confidence interval was estimated using bias-corrected and accelerated nonparametric bootstrap resampling with 20,000 replicates. Individuals without a documented subsequent negative result were not included in this comparison; no observations were treated as censored in this analysis.

For the vaccination analyses, modified Poisson regression with robust standard errors was used to estimate adjusted prevalence ratios (aPRs) and 95% confidence intervals for NAb and IgG positivity after three versus two vaccine doses. Models were adjusted for age, sex, the interval from the most recent vaccine dose to antibody testing, and disease group categorized as hematologic disease, solid tumor, or no documented hematologic disease or solid tumor. Logistic regression was performed as a sensitivity analysis, with results reported as adjusted odds ratios (aORs). Associations between log10-transformed antibody measurements were evaluated using simple linear regression. All tests were two-sided, and *p* < 0.05 was considered statistically significant.

## 3 Results

### 3.1 Recorded RT-PCR positivity among tested individuals was lower in the hematologic disease group

Among the 20,813 individuals who underwent SARS-CoV-2 RT-PCR testing, 2,155 had a recorded positive result. The proportion of tested individuals with a positive result was lower in the hematologic disease group than in the group without hematologic diseases (64/1,341 [4.77%] vs. 2,091/19,472 [10.74%]; unadjusted risk ratio, 0.44; 95% CI, 0.35–0.57; χ^2^ = 48.11, *p* < 0.001). A subsequent negative result was documented for all 64 RT-PCR-positive individuals with hematologic diseases and for 381 of the 2,091 without hematologic diseases. Among these 445 individuals, the median interval to documented RT-PCR negativity was 5.05 days (IQR, 3.26–10.66) and 3.99 days (IQR, 2.95–5.03), respectively. The distributions differed between the groups (Mann–Whitney *U* = 16,668, *p* < 0.001), and the difference in medians was 1.06 days (95% CI, 0.73–2.21; Table 1).

Age-stratified proportions with a recorded positive RT-PCR result among the 1,341 tested individuals with hematologic diseases are shown in Figure 2. RT-PCR positivity was numerically highest among individuals aged 21–40 years (6.38%), followed by those aged 41–60 years (4.66%), 0–20 years (3.12%), 61–80 years (2.75%) and 81–100 years (0%). However, the differences across age groups were not statistically significant (Fisher–Freeman–Halton test, *p* = 0.227). Among the 64 RT-PCR-positive patients with hematologic diseases, three deaths were recorded during follow-up. All three occurred among patients aged 41–60 years, corresponding to a mortality proportion of 13.64% (3/22) in this age group, whereas no deaths were recorded in the other age groups (Table 2).

**Figure 2:**
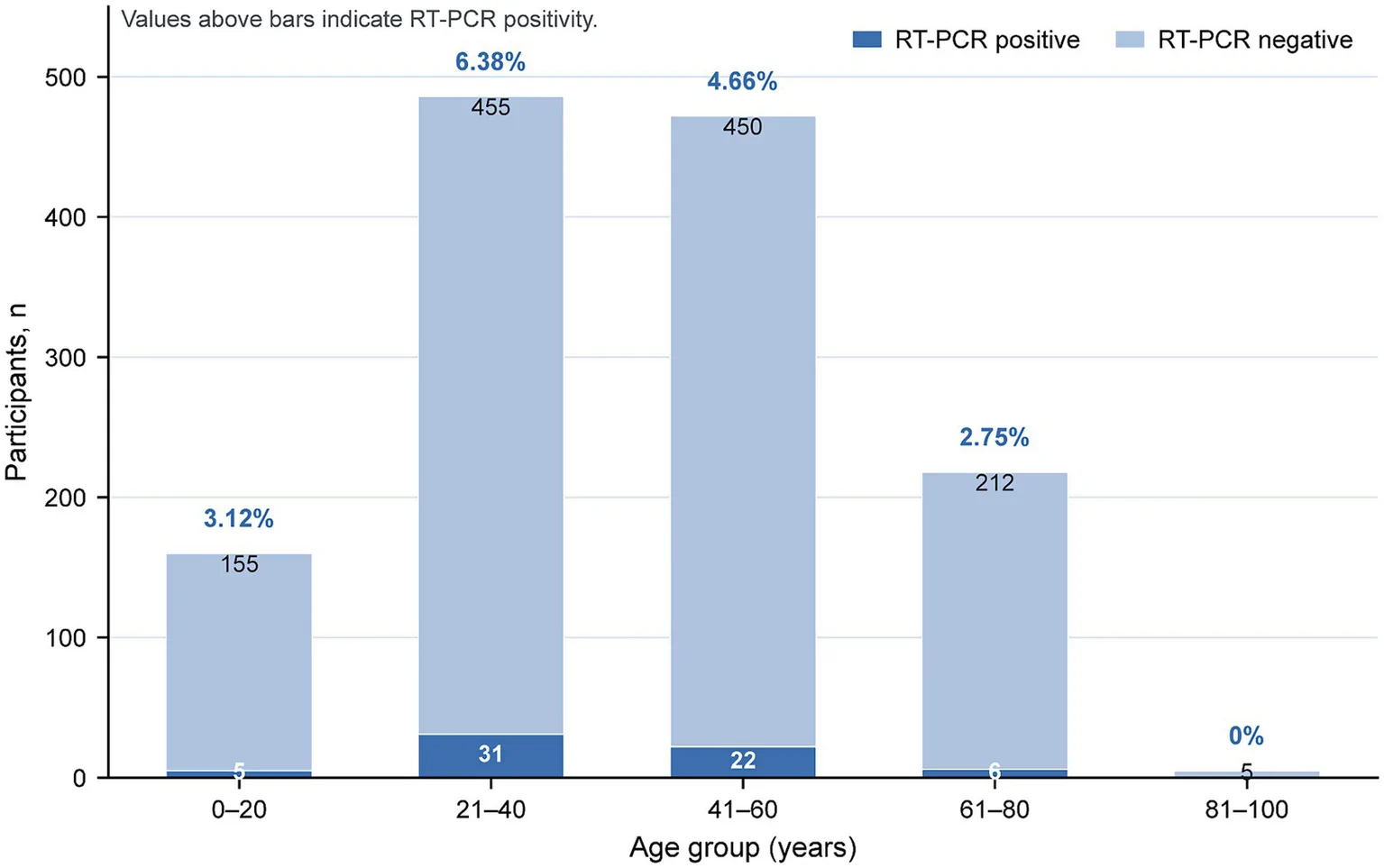
Age-stratified SARS-CoV-2 RT-PCR results among individuals with hematologic diseases. Bars show the numbers of individuals with positive and negative RT-PCR results in each age group. Numbers within or adjacent to the bars indicate participant counts. RT-PCR, reverse-transcription polymerase chain reaction.

### 3.2 Intervals to documented RT-PCR negativity by prior immunosuppressive treatment and Allo-HSCT status

Among the 64 RT-PCR-positive patients with hematologic diseases, the median interval to documented RT-PCR negativity was longest among haploidentical allo-HSCT recipients. At the time of the index positive RT-PCR result, 15 patients had undergone HLA-matched allo-HSCT, 30 had undergone haploidentical allo-HSCT, and 19 had not undergone allo-HSCT. The corresponding median times to RT-PCR negativity were 4.77, 6.97, and 4.00 days, respectively, with the longest median time observed among haploidentical allo-HSCT recipients. Three deaths were recorded during follow-up. Two occurred among haploidentical allo-HSCT recipients, whereas the remaining death occurred in a patient who had not undergone allo-HSCT before infection but subsequently received HLA-matched allo-HSCT. Because only three deaths occurred, the mortality findings were considered descriptive (Table 2).

A longer median interval to documented RT-PCR negativity was also observed among patients who had received immunosuppressive treatment before SARS-CoV-2 infection. Prior immunosuppressive treatment was reported in 46 of the 64 patients (71.88%), whereas 18 (28.13%) had not received immunosuppressive treatment before SARS-CoV-2 infection. The median interval to documented RT-PCR negativity was modestly longer among patients with prior immunosuppressive treatment than among those without prior immunosuppressive treatment (5.99 days; IQR, 4.03–11.35 vs. 3.97 days; IQR, 2.93–5.32; absolute difference, 2.02 days; *p* = 0.010; Table 3).

### 3.3 Anti-SARS-CoV-2 IgG positivity was numerically lower in individuals with hematologic diseases

Among the 2,438 individuals who underwent serum anti-SARS-CoV-2 IgG testing, 2,194 (90.00%) had a positive result. IgG positivity was numerically lower among individuals with hematologic diseases than among those without hematologic diseases (86/100; 86.00% vs. 2,108/2,338; 90.16%), corresponding to an absolute difference of 4.16 percentage points. However, this between-group difference did not reach statistical significance (χ^2^ = 1.845, *p* = 0.174; Table 4).

**Table 4:** Anti-SARS-CoV-2 IgG positivity according to hematologic disease status in the IgG-testing population.

Hematologic disease status	Total, n	IgG positive, *n* (%)	IgG negative, *n* (%)	*χ^2^*	*p*-value
Hematologic disease	100	86 (86)	14 (14)	1.845	0.174
No hematologic disease	2,338	2,108 (90.16)	230 (9.84)		
overall	2,438	2,194 (90.00)	244 (10.00)		

Data are presented as *n* (%). Percentages were calculated using the total number of individuals in each row as the denominator. The P value was calculated using Pearson’s χ^2^ test. IgG, immunoglobulin G.

### 3.4 Antibody positivity remained higher after three versus two doses following multivariable adjustment

The vaccination and antibody-testing population comprised 1,946 participants, including 1,092 who had received two doses and 854 who had received three doses of an inactivated SARS-CoV-2 vaccine (Figure 1). Their demographic and vaccination characteristics and antibody responses are summarized in Table 5. Participants in the three-dose group were older than those in the two-dose group (44.74 ± 14.56 vs. 36.50 ± 12.49 years, *p* < 0.001), whereas the sex distribution was comparable between the groups (*p* = 0.113).

**Table 5:** Demographic and vaccination characteristics and antibody responses according to the number of vaccine doses received.

Characteristic	Two-dose group (*n* = 1,092)	Three-dose group (*n* = 854)	Overall (*n* = 1,946)	*p-*value
Demographic characteristics				
Age, (mean ± SD)	36.50 ±12.49	44.74 ± 14.56	40.11 ± 14.04	<0.001
Age group, *n* (%)				
18–40 years	737 (67.5)	386 (45.2)	1,123 (57.7)	
41–60 years	311 (28.5)	345 (40.4)	656 (33.7)	
Age >60 years	44 (4.0)	123 (14.4)	167 (8.6)	
Sex, *n* (%)				0.113
Male	524 (48.0)	379 (44.4)	903 (46.4)	
Female	568 (52.0)	475 (55.6)	1,043 (53.6)	
Antibody response, *n* (%)				
NAbs positive	844 (77.3)	804 (94.1)	1,648 (84.7)	<0.001
Anti-SARS-CoV-2 IgG positive	911 (83.4)	830 (97.2)	1741 (89.5)	<0.001
Anti-SARS-CoV-2 IgM or IgG positive	1,019 (93.3)	830 (97.2)	1849 (95.0)	<0.001
Vaccination characteristics				
Interval between two most recent vaccine doses (days)	21 (0–77)	210 (7–410)	/	
Interval between antibody test date and the most recent vaccine dose (median days)	50 (0–332)	60.5 (1–379)	/	

The interval refers to the first-to-second dose interval in two-dose recipients and the second-to-third dose interval in three-dose recipients. SD, standard deviation.

NAb positivity was higher in the three-dose group than in the two-dose group (804/854; 94.1% vs. 844/1,092; 77.3%; absolute difference, 16.8 percentage points; *χ^2^* = 104.99, *p* < 0.001). IgG positivity was also higher in the three-dose group (830/854; 97.2% vs. 911/1,092; 83.4%; *p* < 0.001). Similarly, positivity for either anti-SARS-CoV-2 IgM or IgG was higher after three doses than after two doses (830/854; 97.2% vs. 1,019/1,092; 93.3%; *p* < 0.001) (Table 5). After adjustment for age, sex, the interval from the most recent vaccine dose to antibody testing, and disease group, receipt of three doses remained associated with higher NAb positivity (aPR, 1.281; 95% CI, 1.232–1.332; *p* < 0.001) and IgG positivity (aPR, 1.229; 95% CI, 1.189–1.271; *p* < 0.001; Table 6). Logistic regression sensitivity analyses yielded consistent findings (Supplementary Table S1).

**Table 6:** Adjusted associations between receipt of three vaccine doses and antibody positivity.

Outcome	Vaccine doses	Positive/Total	Positivity, %	Adjusted prevalence ratio	95% CI	*p*-value
NAb positivity	Two doses	844/1,092	77.3	Reference		—
	Three doses	804/854	94.1	1.281	1.232–1.332	<0.001
IgG positivity	Two doses	911/1,092	83.4	Reference		
	Three doses	830/854	97.2	1.229	1.189–1.271	<0.001

In the cross-sectional analysis stratified by time since the most recent vaccine dose, NAb positivity in the two-dose group was 82% at month 1 and 88% at month 2; the corresponding cross-sectional estimates from months 3 to 7 were 76%, 61%, 69%, 46%, and 33%, respectively. The month-8 estimate of 100% was based on only one participant and was therefore not considered informative. In the three-dose group, cross-sectional NAb positivity ranged from 86 to 100% across the examined post-vaccination intervals (Figure 3). However, estimates at the later time intervals were based on relatively small numbers of participants. Because the monthly categories comprised different participants, these findings describe population-level patterns associated with time since vaccination rather than within-participant longitudinal changes.

**Figure 3:**
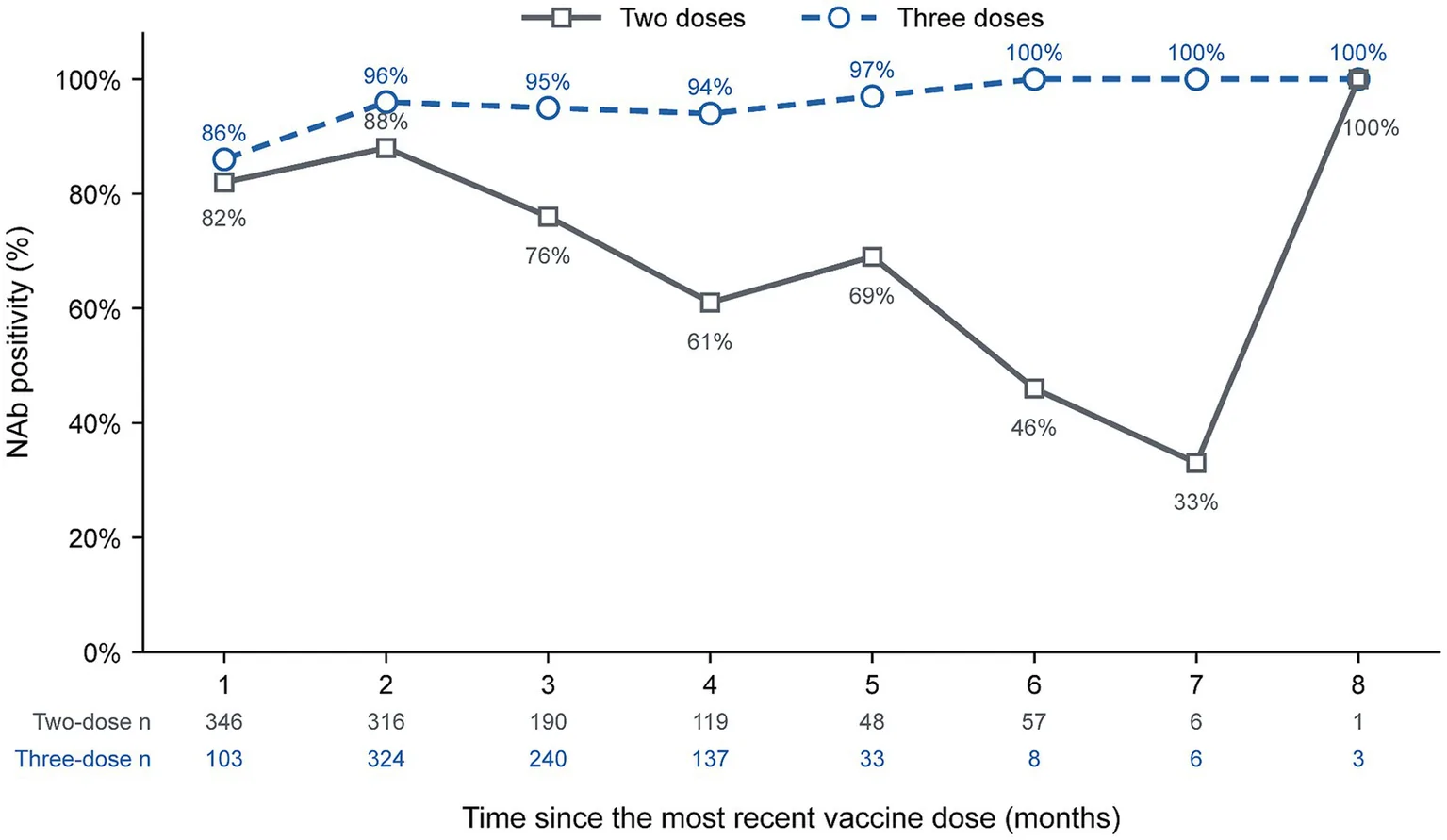
NAb positivity according to time since the second or third vaccine dose. Points indicate the percentage of participants with a positive NAb result within each monthly category. The numbers below the horizontal axis indicate the numbers tested in the two-dose and three-dose groups, respectively. Monthly categories comprised different participants tested at different post-vaccination intervals. NAb, neutralizing antibody.

A nested repeated-measures subcohort comprised 171 participants with serial antibody measurements obtained during the second- and/or third-dose periods (Figures 1, 4). Because the sampling schedules differed among participants, direct comparisons between the second- and third-dose periods were restricted to those with paired measurements available at both periods. Among 20 participants with paired measurements obtained after the second and third doses, 19 (95.0%) showed higher NAb levels after the third dose. This exploratory within-participant finding was consistent with a higher NAb response after the third dose, although the paired sample was small.

**Figure 4:**
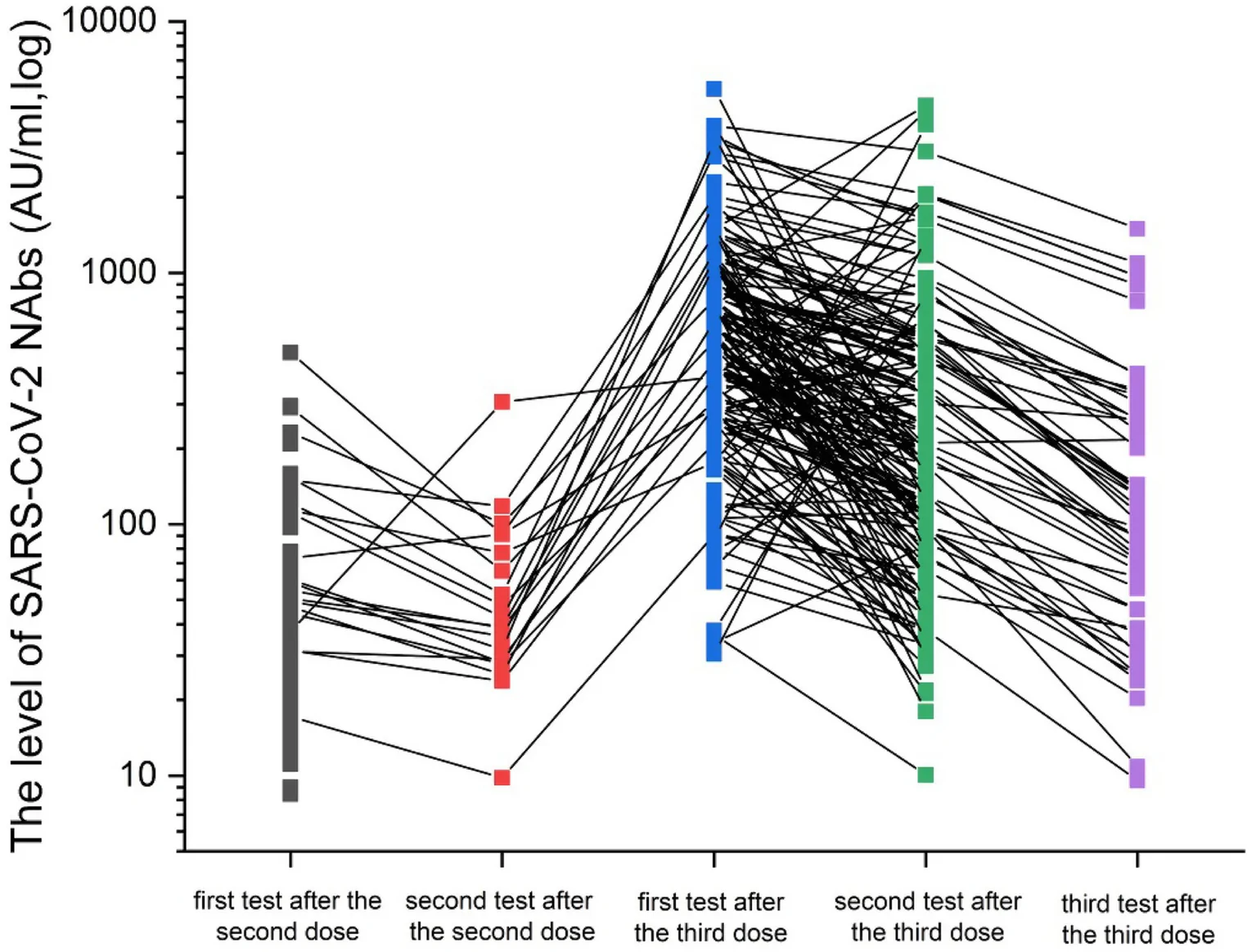
Serial within-participant changes in NAb levels after the second and third vaccine doses. Each line connects available measurements obtained from the same participant. Participants did not necessarily contribute measurements at every assessment point. Only 20 participants had measurements after both the second and third doses and contributed to the direct paired comparison. NAb levels are displayed on a logarithmic scale.

### 3.5 NAb positivity was high across months 1–5 after the third dose in recipients with a dose interval of ≥180 days

Among the 854 three-dose recipients, 810 had an interval of ≥180 days between the second and third doses, whereas 44 had an interval of <180 days. In the ≥180-day group, NAb positivity was 86% at month 1, 96% at month 2, 95% at months 3 and 4, and 97% at month 5 after the third dose. NAb positivity was 100% at months 6 and 7; however, these estimates were based on only one and two participants, respectively, and were therefore not considered informative (Figure 5).

**Figure 5:**
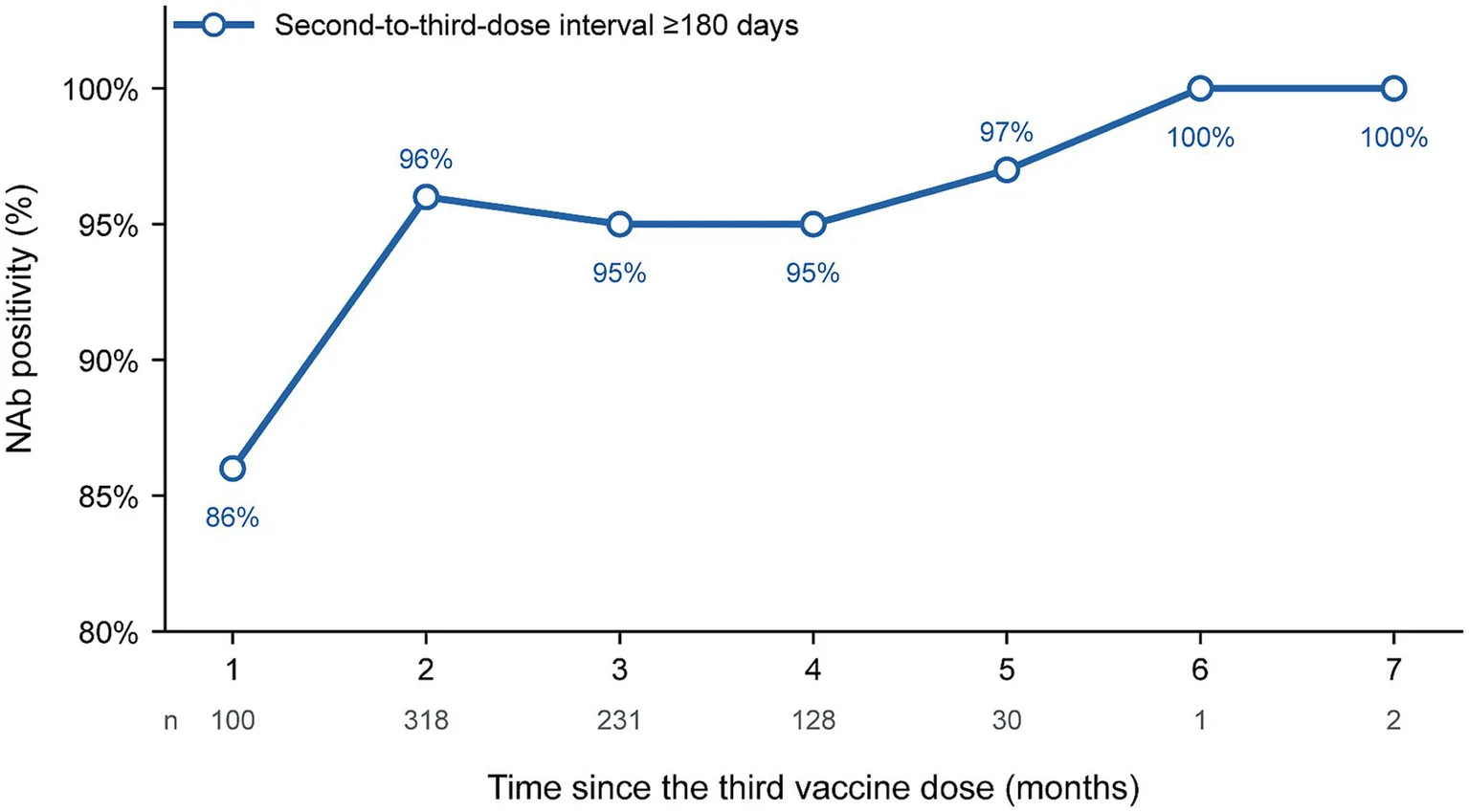
NAb positivity after the third vaccine dose among recipients with a second-to-third-dose interval of ≥180 days. Points indicate the percentage of participants with a positive NAb result within each monthly category after the third dose. The numbers below the horizontal axis indicate the number of participants tested in each category. NAb, neutralizing antibody. Monthly categories comprised different participants.

Cross-sectional NAb levels were predominantly above the assay positivity threshold in both vaccination-interval groups, although substantial interindividual variability was observed within each monthly category (Figures 6, 7). NAb levels did not show a uniform monotonic pattern across the post-third-dose time categories. Estimates at the later intervals were interpreted cautiously because they were based on relatively few participants, particularly in the <180-day group.

**Figure 6:**
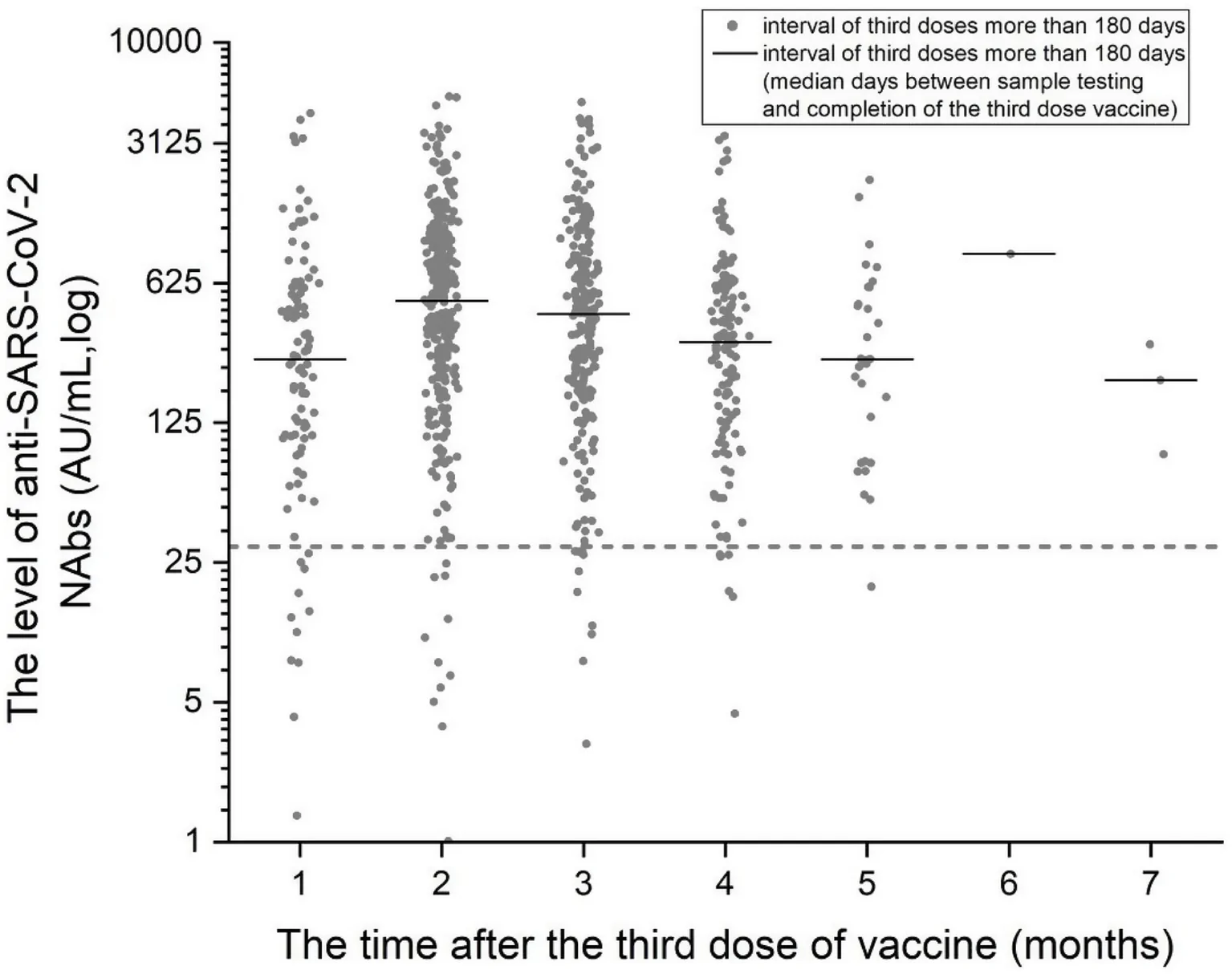
Distribution of NAb levels after the third vaccine dose among recipients with a second-to-third-dose interval of ≥180 days. Each point represents one participant within the corresponding monthly category. Horizontal solid lines indicate median NAb levels. The vertical axis is displayed on a logarithmic scale.

**Figure 7:**
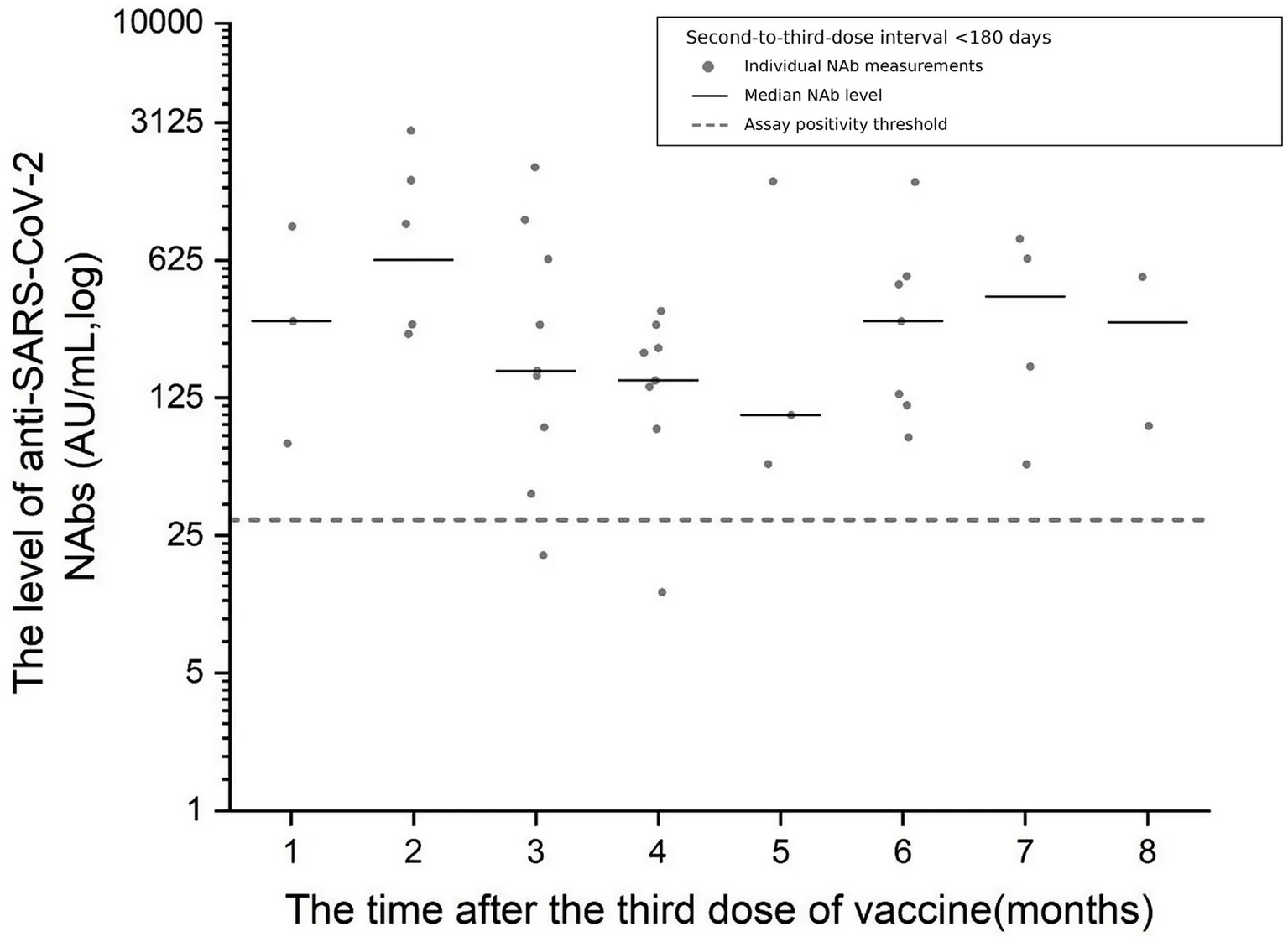
Distribution of NAb levels after the third vaccine dose among recipients with a second-to-third-dose interval of <180 days. Each point represents one participant within the corresponding monthly category. Horizontal solid lines indicate median NAb levels. The vertical axis is displayed on a logarithmic scale.

### 3.6 NAb positivity was lower among three-dose recipients with hematologic diseases

Among three-dose recipients, NAb positivity was 25/32 (78.13%) among participants with hematologic diseases, 36/42 (85.71%) among those with solid tumors, and 743/780 (95.26%) among those without documented hematologic disease or solid tumors (overall *p* < 0.001; Table 7; Figure 8). Compared with participants without documented hematologic disease or solid tumors, NAb positivity was significantly lower among those with hematologic diseases (*p* = 0.001). Findings for the solid-tumor subgroup were considered exploratory because of the limited sample size.

**Table 7:** NAb positivity among three-dose recipients according to disease group.

Disease group	Total	NAb positive, *n* (%)	NAb negative, *n* (%)	χ^2^	*p*-value
Hematologic disease	32	25 (78.13)	7 (21.88)		
Solid tumor	42	36 (85.71)	6 (14.29)		
Reference group	780	743 (95.26)	37 (4.74)		
Overall	854	804 (94.15)	50 (5.85)	22.063	<0.001

The reference group comprised participants without documented hematologic disease or solid tumors. NAb, Neutralizing antibody.

**Figure 8:**
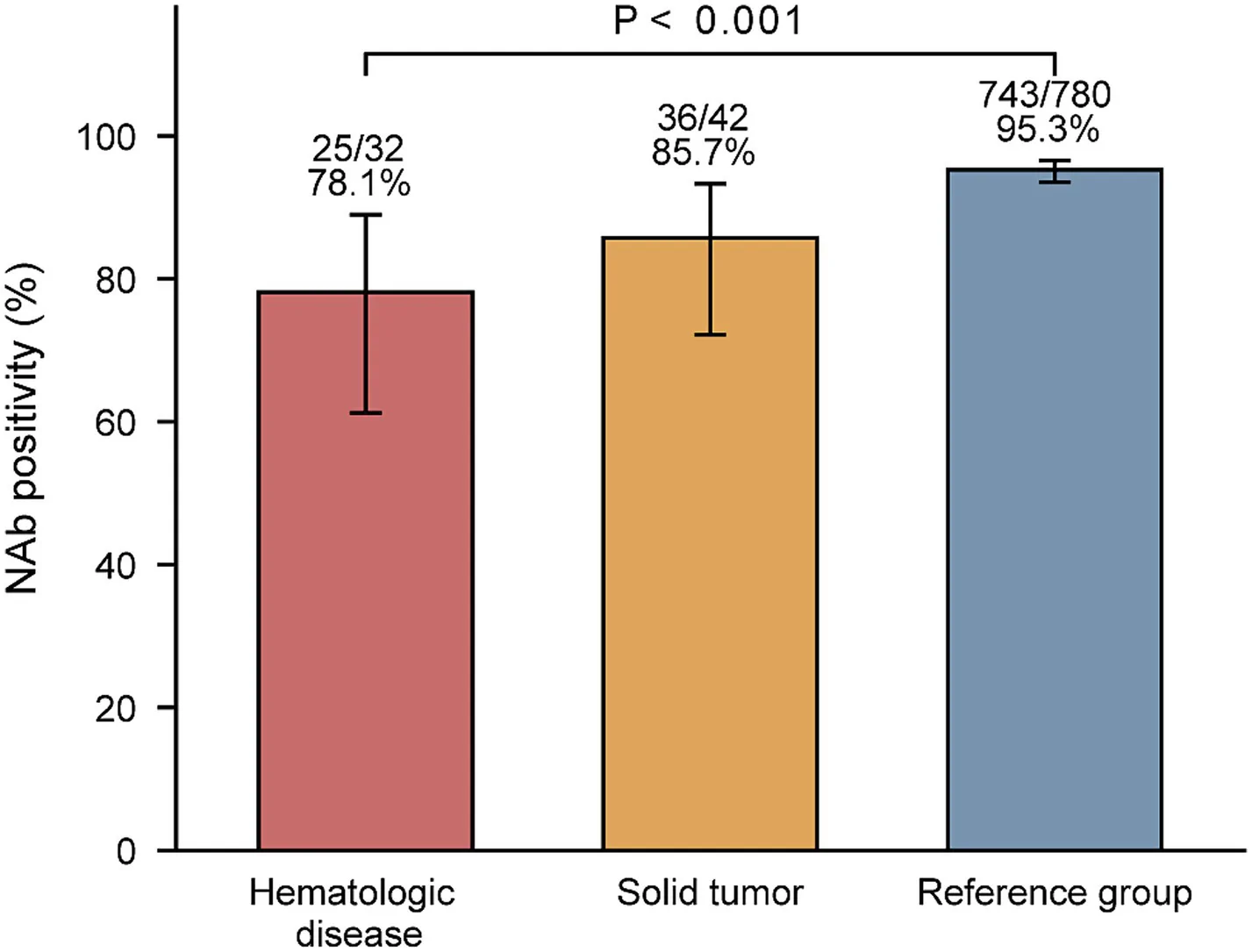
NAb positivity among three-dose recipients according to disease group. Bars show the percentage of participants with a positive NAb result in the hematologic disease, solid-tumor, and reference groups. Labels indicate the number of participants with positive results and the total number tested.

### 3.7 NAb levels were strongly associated with anti-SARS-CoV-2 IgM or IgG measurements

Linear regression analysis showed a positive association between log-transformed NAb levels and log-transformed anti-SARS-CoV-2 IgM or IgG signal-to-cutoff values (*y* = 0.68*x* − 0.45, *R*^2^ = 0.83, *p* < 0.01) (Figure 9). NAb positivity was higher among participants who were positive for either anti-SARS-CoV-2 IgM or IgG than among those who were negative for both antibodies (96.7% vs. 4.2%, *p* < 0.001). These findings demonstrated concordance between NAb results and the other SARS-CoV-2 antibody measurements.

**Figure 9:**
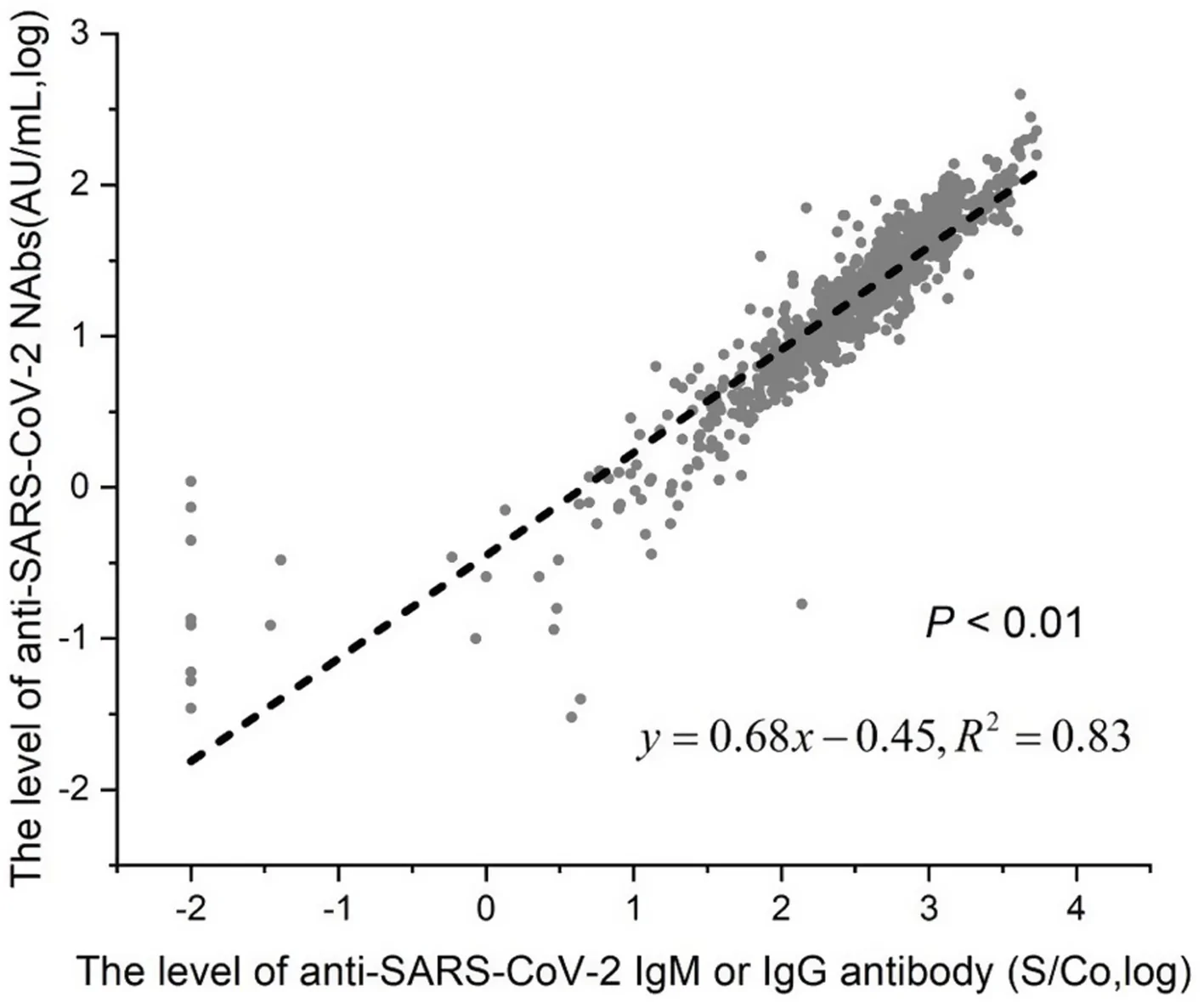
Association between NAb levels and anti-SARS-CoV-2 antibody measurements. The scatterplot shows log10-transformed NAb levels plotted against log10-transformed IgG or IgM S/CO values. The dashed line represents the fitted simple linear regression line (*y* = 0.68x − 0.45; *R*^2^ = 0.83; *p* < 0.01).

## 4 Discussion

Based on three independently identified real-world populations at Peking University People’s Hospital, this retrospective study evaluated SARS-CoV-2 RT-PCR outcomes and humoral immune responses. Among those tested by RT-PCR, individuals with hematologic diseases had a lower proportion of recorded positive results. Among RT-PCR-positive individuals with a documented subsequent negative result, the interval to documented negativity was modestly longer in the hematologic disease group. Within this group, a longer interval was observed among patients with prior immunosuppressive treatment, while haploidentical allo-HSCT recipients had the longest descriptive median interval among the transplantation subgroups. In the separate vaccination population, three doses were associated with higher NAb and IgG positivity after multivariable adjustment. This study adds real-world evidence on antibody responses following inactivated vaccination and descriptive comparisons of intervals to documented RT-PCR negativity across allo-HSCT subgroups.

The lower proportion of recorded positive RT-PCR results among tested individuals with hematologic diseases was unexpected. Because testing arose from routine clinical care, differences in testing indications, exposure, healthcare contact, and protective behavior may have influenced this comparison. It should therefore not be interpreted as a difference in infection incidence or susceptibility.

Among RT-PCR-positive individuals with a documented subsequent negative result, the median interval to documented negativity was modestly longer in the hematologic disease group. Impaired antiviral responses may have contributed to this finding, consistent with reports of prolonged SARS-CoV-2 persistence in some immunocompromised patients (23, 24). However, a subsequent negative result was available for all 64 RT-PCR-positive individuals with hematologic diseases but for only 381 of 2,091 without hematologic diseases, and differential follow-up may therefore have influenced the comparison. Although statistically significant, the difference in medians was modest at 1.06 days (95% CI, 0.73–2.21), and its clinical importance remains uncertain because no established threshold defines a clinically meaningful difference in this outcome.

Stratification by allo-HSCT status showed that haploidentical allo-HSCT recipients had the longest median interval to RT-PCR negativity. This finding may reflect delayed immune reconstitution, graft-versus-host disease, or continued immunosuppressive treatment. Two of the three deaths occurred among haploidentical allo-HSCT recipients, but the small number of events precludes conclusions regarding transplantation type and mortality. Previous studies have similarly reported adverse COVID-19 outcomes among HSCT recipients (25). The longer observed median interval among haploidentical allo-HSCT recipients is descriptive. Small subgroup sizes, differences in treatment, and the timing of repeat testing prevent a firm conclusion about the effect of transplant type on RT-PCR negativity.

All three deaths occurred among patients aged 41–60 years, whereas no deaths were recorded in the other age groups. This differs from European EPICOVIDEHA data identifying advanced age as a significant predictor of COVID-19 mortality in hematologic cancer patients (26). However, only three deaths occurred in our study, and treatment intensity and transplantation status may have differed across age groups. This finding should therefore be considered descriptive rather than evidence of increased mortality risk among middle-aged patients.

In the separate IgG-testing population, IgG positivity was numerically lower among individuals with hematologic diseases, but the difference was not statistically significant. Because infection and vaccination histories were not uniformly available, the source and durability of these antibodies could not be determined. Nevertheless, impaired B-cell function associated with hematologic diseases and their treatments provides a plausible explanation for reduced antibody responses. Previous studies have also reported lower SARS-CoV-2 antibody positivity and reduced vaccine seroconversion among patients with hematologic malignancies (27, 28).

Booster vaccination is particularly relevant to patients with impaired immunity. In the primary vaccination population, the associations of three doses with higher NAb and IgG positivity persisted after adjustment for age, sex, time from the most recent vaccine dose to antibody testing, and disease group. This finding is consistent with previous evidence that booster vaccination enhances humoral responses following inactivated vaccines (29–32). Among 20 paired participants, 19 had higher NAb levels after the third dose; this finding should be considered exploratory because of the small sample size.

The analyses according to time since vaccination should be interpreted cautiously. The monthly categories comprised different participants, and the later categories contained very small samples. The serially tested subgroup also had nonuniform sampling schedules and heterogeneous antibody trajectories. Consequently, the present study could not define the duration or rate of antibody decline after the third dose. Although antibody waning following inactivated vaccination has been reported previously (33–35), standardized longitudinal studies are required to characterize long-term antibody kinetics in patients with hematologic diseases.

Antibody responses were also examined across disease groups. NAb positivity was lower among participants with hematologic diseases than among those without documented hematologic disease or solid tumors (*p* = 0.001). Results for the solid-tumor subgroup should be considered exploratory because of its limited sample size. Previous studies have reported impaired vaccine responses following B-cell-depleting or prolonged immunosuppressive treatment (36, 37), although substantial antibody increases after booster vaccination have also been observed in some cancer populations (38). Because antibody positivity does not directly indicate clinical protection, further studies are needed to determine optimal vaccination and monitoring strategies for patients with hematologic diseases. The overall vaccination findings primarily reflect participants without documented hematologic disease or solid tumors and should not be directly generalized to patients with hematologic diseases. The hematologic disease subgroup comprised only 32 three-dose recipients, limiting the precision and generalizability of its findings.

This study has several limitations. First, its single-center retrospective design and the limited sample sizes of certain subgroups, particularly fatal cases and individual hematologic disease subtypes, restricted further multivariable analyses. In addition, the comparison of intervals to documented RT-PCR negativity included only individuals with an available subsequent negative result and may therefore have been affected by differential follow-up and selection bias. Second, although the vaccination models adjusted for age, sex, testing interval, and disease group, treatment-related variables and detailed measures of immunosuppression were unavailable. This limited subgroup characterization and left potential residual confounding. In addition, the cross-sectional monthly comparisons could not establish within-participant antibody kinetics, and the paired second- versus third-dose analysis included only 20 participants. Finally, the study focused primarily on humoral immunity and did not systematically evaluate cellular immune responses. Future multicenter prospective studies incorporating standardized longitudinal sampling, cellular immunity, viral variants, and long-term clinical outcomes are warranted to further inform vaccination strategies for patients with hematologic diseases.

Overall, among RT-PCR-positive individuals with a documented subsequent negative result, the interval to documented negativity was modestly longer in those with hematologic diseases. Within the hematologic disease group, a longer interval was observed in patients with prior immunosuppressive treatment, while haploidentical allo-HSCT recipients had the longest median interval among the transplantation subgroups. A third dose of inactivated vaccine was associated with higher NAb and IgG positivity, and most paired participants showed increased NAb levels after the third dose. These findings support closer virological and serological monitoring while highlighting the need for prospective studies of vaccination strategies in patients with hematologic diseases.

## Data Availability

The original contributions presented in the study are included in the article/Supplementary material, further inquiries can be directed to the corresponding authors.
