# Supplementary material for: Study on the clinical outcomes of COVID-19 infection and immune responses following the third booster dose of inactivated COVID-19 vaccine in hematologic disease patients
Source: Front Public Health. 2026 Oct 27;14:1917493. doi: 10.3389/fpubh.2026.1917493 (PMC13748446; doi:10.3389/fpubh.2026.1917493)
Supplement: Supplementary file 1 [file Table_1.docx]

Supplementary Table S1. Multivariable regression analyses of factors associated with SARS-CoV-2 antibody positivity

Table S1.1. Modified Poisson regression with robust standard errors

| **Variable** | **NAb positivity, aPR** | **95% CI** | ***P* value** | **IgG positivity, aPR** | **95% CI** | ***P* value** |
| --- | --- | --- | --- | --- | --- | --- |
| Three vs. two vaccine doses | 1.281 | 1.232–1.332 | <0.001 | 1.229 | 1.189–1.271 | <0.001 |
| Age, per 10-year increase | 0.965 | 0.952–0.978 | <0.001 | 0.972 | 0.962–0.982 | <0.001 |
| Male vs. female | 0.957 | 0.922–0.993 | 0.020 | 1.038 | 1.008–1.068 | 0.011 |
| Time from last dose to testing, per 30-day increase | 0.953 | 0.936–0.971 | <0.001 | 0.928 | 0.911–0.944 | <0.001 |
| Hematologic disease vs. reference group | 0.812 | 0.673–0.979 | 0.029 | 0.928 | 0.844–1.020 | 0.123 |
| Solid tumor vs. reference group | 0.928 | 0.820–1.050 | 0.237 | 0.898 | 0.790–1.020 | 0.099 |

Table S1.2. Logistic regression sensitivity analysis

| **Variable** | **NAb positivity, aOR** | **95% CI** | ***P* value** | **IgG positivity, aOR** | **95% CI** | ***P* value** |
| --- | --- | --- | --- | --- | --- | --- |
| Three vs. two vaccine doses | 7.882 | 5.396–11.514 | <0.001 | 14.120 | 8.149–24.465 | <0.001 |
| Age, per 10-year increase | 0.771 | 0.698–0.851 | <0.001 | 0.734 | 0.649–0.831 | <0.001 |
| Male vs. female | 0.707 | 0.544–0.918 | 0.009 | 1.473 | 1.063–2.041 | 0.020 |
| Time from last dose to testing, per 30-day increase | 0.788 | 0.729–0.853 | <0.001 | 0.612 | 0.557–0.672 | <0.001 |
| Hematologic disease vs. reference group | 0.160 | 0.064–0.401 | <0.001 | 0.213 | 0.046–0.994 | 0.049 |
| Solid tumor vs. reference group | 0.363 | 0.142-0.929 | 0.035 | 0.159 | 0.056-0.454 | <0.001 |

NAb positivity was defined as NAb ≥30 and IgG positivity as IgG ≥1.0. Modified Poisson regression with robust standard errors was used to estimate aPRs, and logistic regression was used to estimate aORs. Models were adjusted for age, sex, time from the last vaccine dose to antibody testing, and disease group. Reference categories were two doses, female sex, and no documented hematologic disease or solid tumor. Age and testing time are reported per 10-year and 30-day increases, respectively. aPR, adjusted prevalence ratio; aOR, adjusted odds ratio; CI, confidence interval; NAb, neutralizing antibody.
